# Supplementary figures and images for: A model-based evaluation of the efficacy of COVID-19 social distancing, testing and hospital triage policies
Source: PLoS Comput Biol. 2020 Oct 15;16(10):e1008388. doi: 10.1371/journal.pcbi.1008388 (PMC7591016; doi:10.1371/journal.pcbi.1008388)

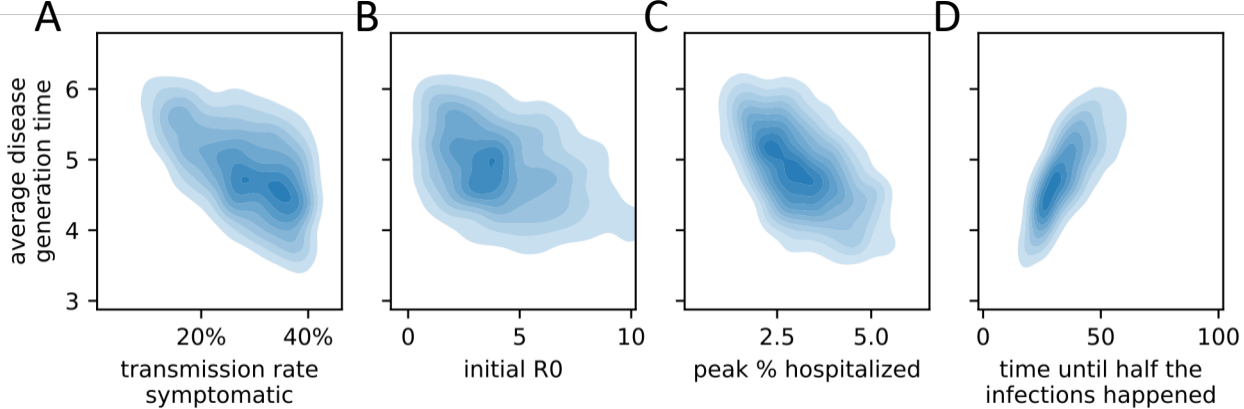

Supplement: S1 Fig — All model parameters were chosen as described in Tables 1 and 2 and in the third column of S1 Table. Bivariate Gaussian kernel density estimates are shown. (PDF) [file pcbi.1008388.s001.pdf]

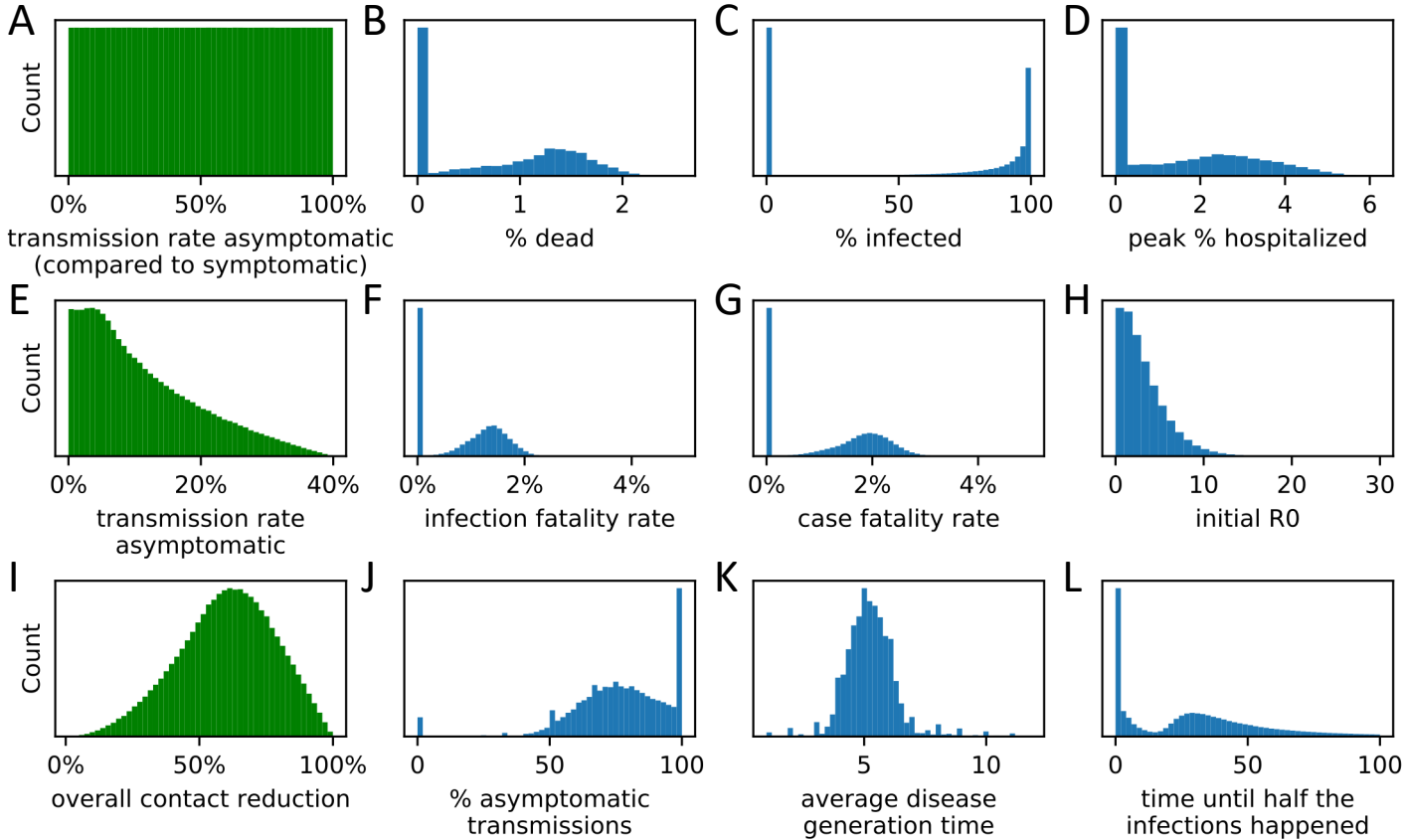

Supplement: S2 Fig — All model parameters were chosen as described in Tables 1 and 2 and in the third column of S1 Table. (PDF) [file pcbi.1008388.s002.pdf]

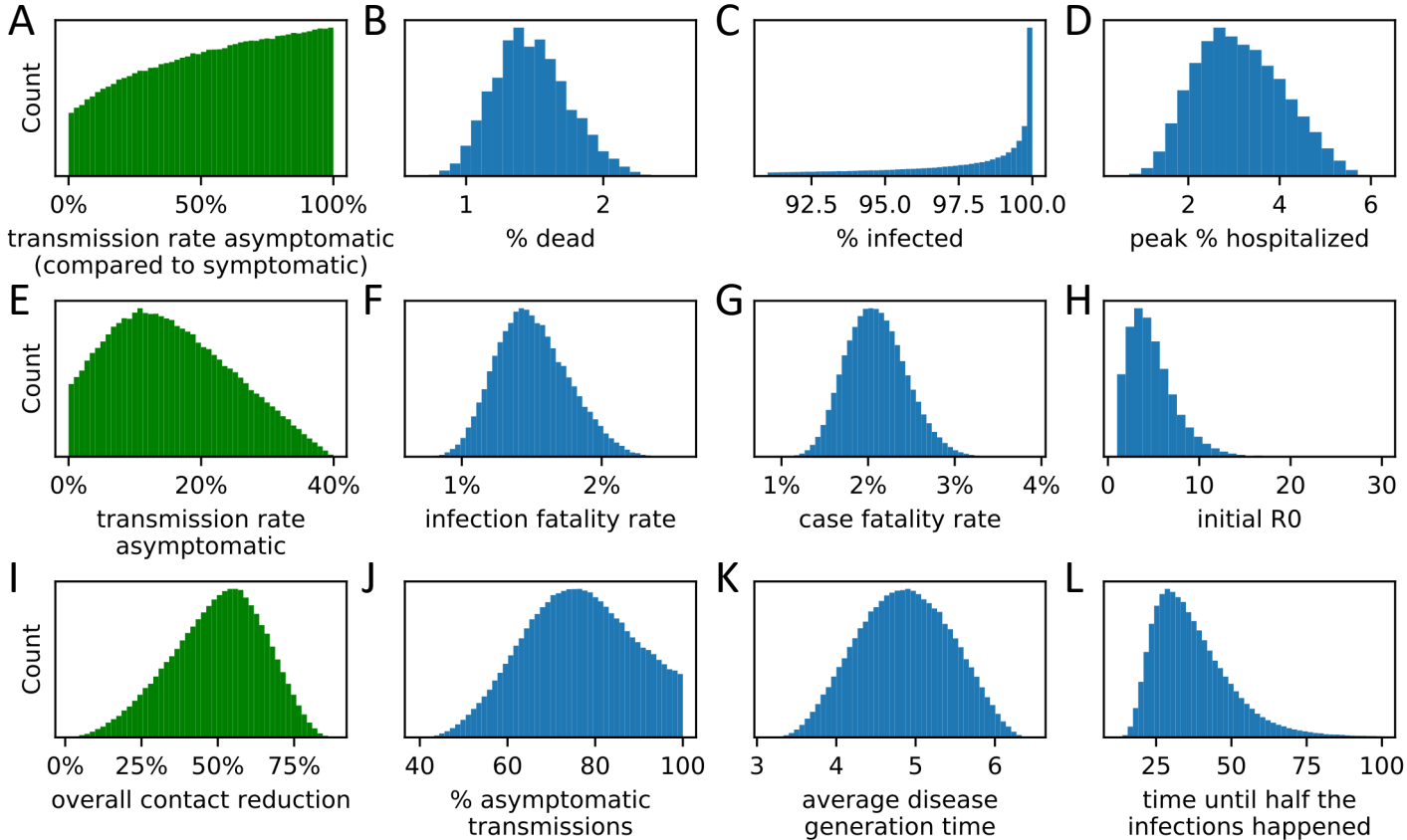

Supplement: S3 Fig — All model parameters were chosen as described in Tables 1 and 2 and in the third column of S1 Table. Data were restricted to those model runs that resulted in at least 10% infected (63.7% of model runs). (PDF) [file pcbi.1008388.s003.pdf]

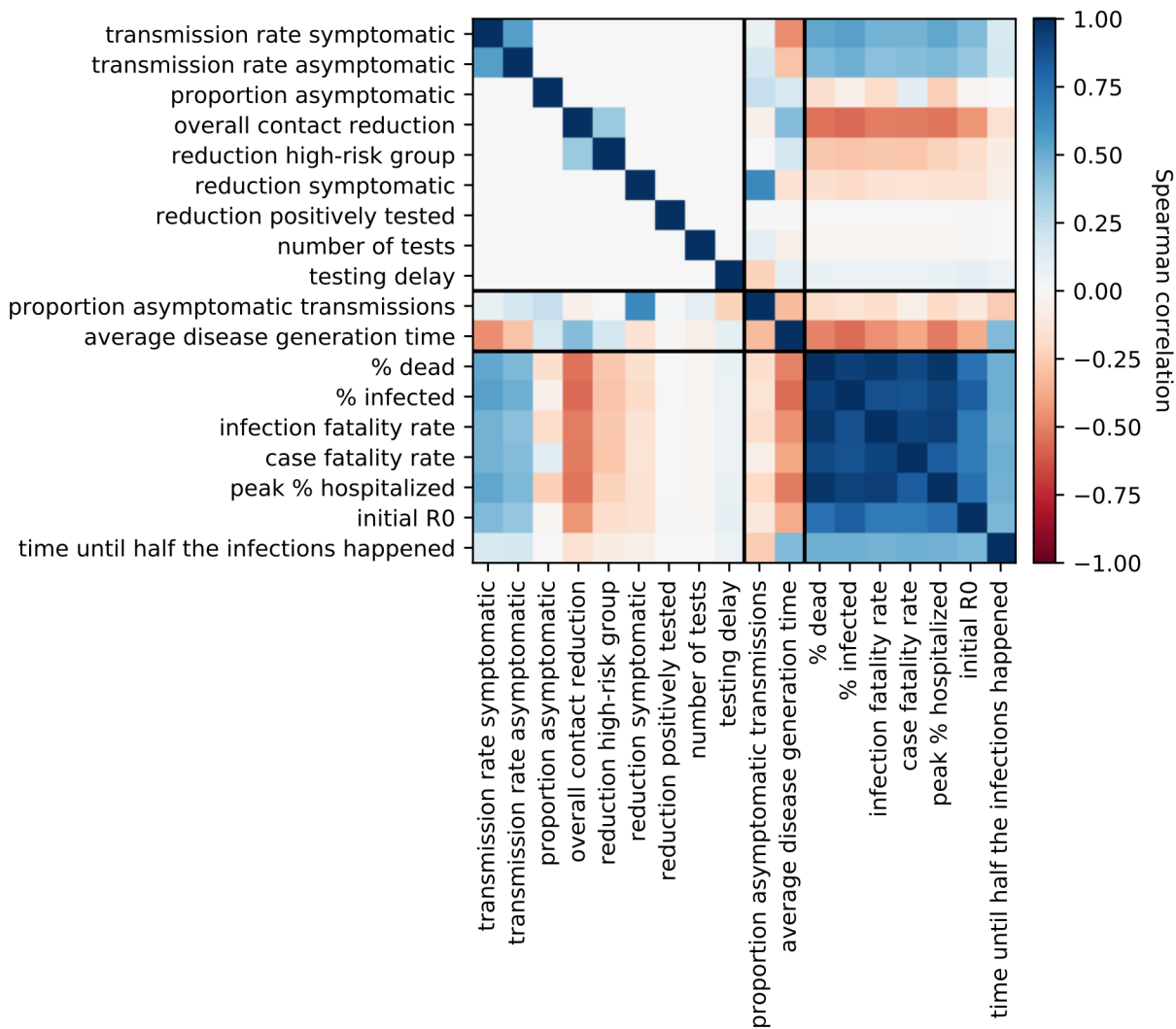

Supplement: S4 Fig — All model parameters were chosen as described in Tables 1 and 2 and in the third column of S1 Table. (PDF) [file pcbi.1008388.s004.pdf]

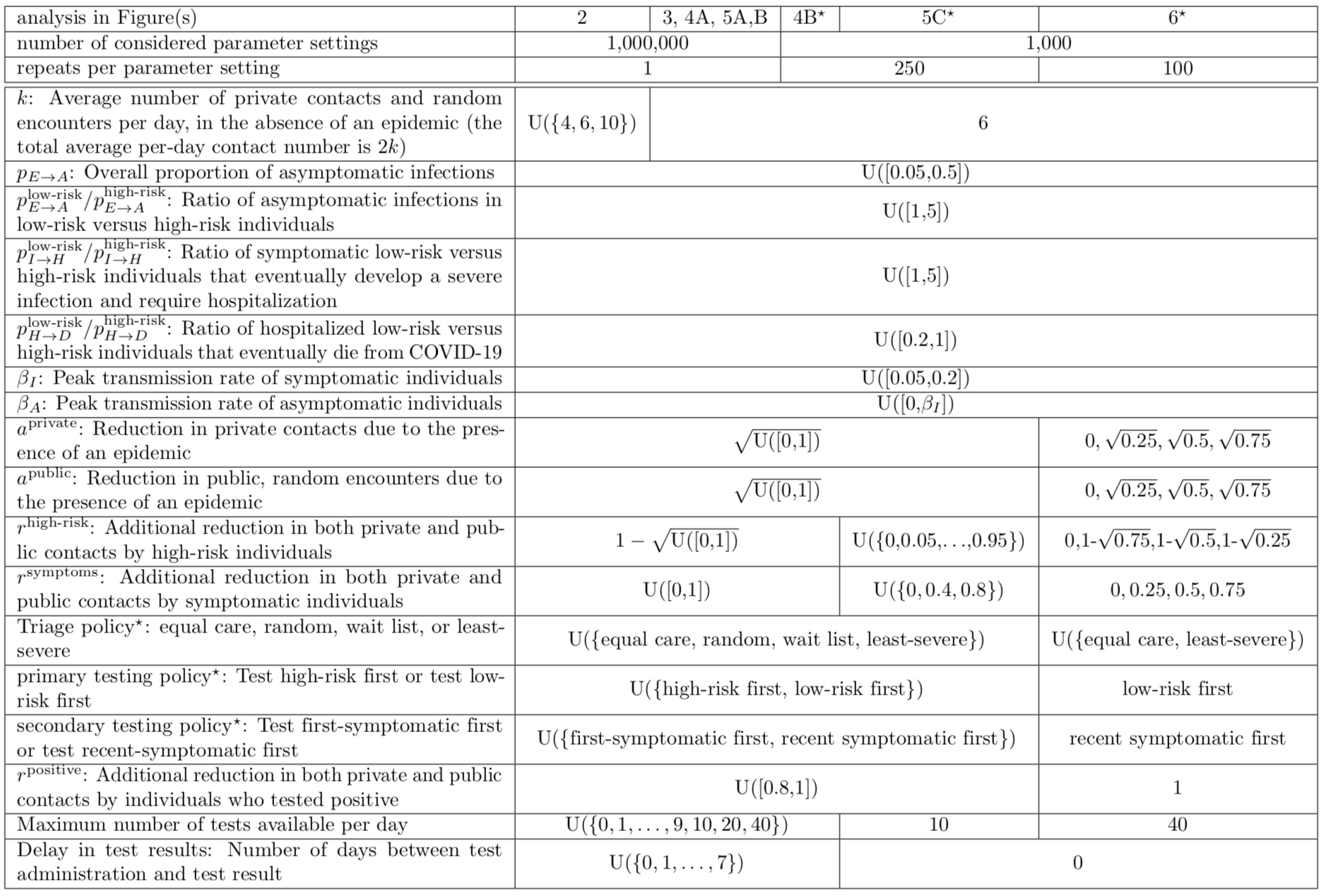

Supplement: S1 Table — Square brackets [] denote sampling from a continuous space, while curly brackets {} denote sampling from a discrete set of values. ⋆ Identical 1,000 parameter settings and identical 250 seeds were used to compare the different triage policies in Fig 4B, and the different combinations of primary and secondary testing policies in Fig 5C. In Fig 6, only 100 seeds were used to compare the different combinations of policy choices. (PNG) [file pcbi.1008388.s005.png]
