# Supplementary material for: A model-based evaluation of the efficacy of COVID-19 social distancing, testing and hospital triage policies
Source: PLoS Comput Biol. 2020 Oct 15;16(10):e1008388. doi: 10.1371/journal.pcbi.1008388 (PMC7591016; doi:10.1371/journal.pcbi.1008388)
Supplement: Attachment — Submitted filename: Response_to_reviewers.pdf [file pcbi.1008388.s006.pdf]

Reviewer #1:

General Comment: A timely model that would be very helpful as a framework for considering complex compartmental models that addresses multiple concerns at once. I would recommend it for publication after minor edits. Currently, in the US, it seems that all of the policies that have been implemented have worked against one another and the public in general seems confused as to what exactly is scientifically acceptable behavior during this difficult time. I think a model like this can help guide future policy implementation and keep the oversaturation of control measures from overwhelming the public.

We thank the reviewer for the positive comment.

Other comments:

Abstract: Line 1 Reword, phrase "SARS-CoV-2 and COVID-". These are not two different diseases. One is the name of the virus and the other is the disease caused by the virus, see line 2.

We thank the reviewer for the comment. This study models both the spread of the virus SARS-CoV-2 through an interaction network, as well as the effects of the severity of the disease COVID-19 on hospital capacity, but we agree: it would e.g. also sound strange to write of a model of HIV and AIDS. We therefore dropped COVID-19 from this sentence and clarify in a later sentence in the abstract that COVID-19 is the disease caused by SARS-CoV-2.

Lines 9-15: May be lead this paragraph with what these policy domains are before saying no modeling efforts have looked at them simultaneously.

We have edited the paragraph for improved readability as the reviewer suggests.

Line 51-52: It would be good to also explore how these policies impact case incidence

We agree with the reviewer. Our model produces results for 9 different outcome measures of virus and disease effects, including case incidence (labelled "% infected" in figures S2 and S3). As we indicate on lines 63-65 of the manuscript, all model outcomes were highly correlated, therefore in reporting our results we focus on COVID-19-related mortality.

Line 53-55: I'm very interested to see how this model fairs with more accurate data and tailored to a specific city.

We agree with the reviewer. As we discuss on lines 150-158 of the manuscript, our model lacks many elements of more realistic social networks, including elements unique to a specific location or city such as population size or per-day contact rates. Our model could be tailored to a specific city in future refinements. Also, as our understanding of SARS-CoV-2 and COVID-19 improves, estimates of the parameters we use in our model will become more accurate, and these improved estimates can certainly be incorporated into future refinements to the model. However, in this initial study, we chose to keep the network structure quite

simple in order to focus on a general understanding of how different policy decisions interact.

Line 74-75: consider adding an ie statement here to make things a bit more clear

We added a clarifying sentence:

The proportion of infections caused by asymptomatic cases was influenced more by the behavior of symptomatic individuals than by the actual rate of asymptomatic cases. That is, a given increase in contact reduction of symptomatic individuals caused a bigger change in the proportion of infections caused by asymptomatic cases than an equal increase in the proportion of asymptomatic cases (Fig.3).

Fig 2: Is it accurate to assume the same number of private and public contacts? This is discussed a bit later, but it would be nice to see the effects of having more/less public interactions for say, a politician, vs an average citizen in the US

We are unaware of explicit data regarding the number of "private" vs "public" contacts. However, we agree with the reviewer that the inclusion of complicating elements of more realistic social networks, such as the inclusion of super-spreaders and variability in the number of private and public contacts, would be interesting. For this study we were concerned with keeping the number of model parameters manageable in order to compare the interactions between different policy domains. More realistic elements of social networks would increase the number of model parameters substantially and necessitate further sensitivity analyses.

We also added additional sentences in the Discussion where we list possible extensions, in particular regarding heterogeneity in the number of contacts, and their potential effects on our findings (lines 166-176).

Fig 3-4: Once everything is formatted, I think this section will flow very well
-It seems like some things could be moved to/from this section and the discussion section. Some explanation of the results is given, but not fully expanded on until the discussion section, which was a bit jarring, but did not retract from my understanding of the content in a major way

We thank the reviewer for the comment.

Line 167: I don't see a Fig C anywhere. Is it fig 1, C? Or in the supplementary materials?

We thank the reviewer for catching this typo. The correct reference should have been to Fig. S2C, and we have revised the text accordingly.

Line 187: seeing this model adapted to a particular school with better parameter estimates to see possible effects of school reopenings would be interesting

We thank the reviewer for the comment, and agree that this would indeed be very interesting. However, as pointed out in response to the comment regarding line 53-55, we specifically

chose a generic, rather simple contact structure to focus on the interactions between different policy decisions. Further refinements and adaptations of this model to investigate the effects of school reopenings are unfortunately beyond the scope of this theoretical study.

Lines 194: "We modeled the spread of an imported case"
This was not mentioned in the main text, not the effect this imported case

This comment seems to be incomplete, however we address the issue of "imported" cases in our response to line 196 below.

Line 195: have you tested your model with different sized populations to see if any of the dynamics change?

Yes. We originally ran the model for a population of 1000, and the results were qualitatively identical to the model with population 10,000. We have added language to indicate this at lines 217-219.

Line 196: closed and imported seem to be contradicting themselves here.

We have removed all language referring to an "imported" case as suggested by the reviewer.

Lines 206-208: I think it's important to include those individuals that properly quarantine and thus have close to 0 contacts per day, especially if a high-risk individual tests positive

The number of contacts, k, referred to in this sentence is the average number of contacts in the absence of an epidemic. We account for a reduction in social contacts due to risk level and positive test results through separate model parameters, $r^{symptoms} \sim U(0,1)$, and $r^{high\text{-}risk}$, $\sim U(0,1)$ (see lines 328-336). These rates are to be interpreted as population-wide averages—some individuals may reduce their interactions more, while others may reduce their interactions less, and the average reduction rate is sampled from a Uniform(0,1) distribution.

Lines 212-214: What is the justification for the selection of these numbers?

We thank the reviewer for pointing out a missing reference here. The justification for these numbers is explained in Table 1. Specifically, our estimate is derived from Koma et al. 2020, "How many adults are at risk of serious illness if infected with Conronavirus." We note in the caption to Table 1 that the referenced study considers only the U.S. adult population (age 18+). Adding 0-17 year olds with the same risk rate as 18-59 year olds yields an overall high-risk estimate around 1/3. We have added a citation to Koma et al. 2020, the US census data as well as a reference to Table 1 for clarification.

Lines 219-220: See above, you simply initialized the model with one infected, the one infected is not due to importation

We revised the language to remove the reference to an imported case:

To model the spread ~~of an imported case~~ of the virus in a fully susceptible population, we

initialized the simulation with one random individual…

Line 222: See my comment above, not two different disease

In the abstract, we agree with the reviewer that it is helpful to explain that SARS-CoV-2 is the virus that causes the disease COVID-19. However, here we are concerned with virus- and disease-specific parameters and believe that it thus makes sense to write “the specific characteristics of SARS-CoV-2 and COVID-19” just like one would write “the specific virus and disease parameters”.

Line 248-252: it would be interesting to explore the possibility that asymptotic individuals having a higher transmission rate

We thank the reviewer for this comment; however, we have seen no evidence in the literature that asymptomatic individuals could have a higher transmission rate. If the reviewer is aware of studies that suggest this, we would be grateful for further direction and inclusion of this expanded parameter space for the asymptomatic transmission rate. Because we have found no evidence for a higher transmission rate in asymptomatic individuals, to keep the considered parameter spaces as realistic as possible we capped the asymptomatic transmission rate at the rate of symptomatic transmission.

Line 237: Remove parenthesis on the statement (and transition to the exposed compartment E) them move it to line 238 after “contagious individuals”

We revised the text as suggested by the reviewer.

Line 268-269: It is possible in less well-developed countries that infected individuals could die before being hospitalized, so this ratio may need to be adjusted to reflect that. It will be good to may a statement about this in the text.

Many of the assumptions of the model, including assumptions about testing and the structure of the social network, are based on the circumstances present in developed countries, where the pandemic spread fastest in its early phase. We included the best available data on virus and disease parameters from these areas. We do agree with the reviewer, however, that assumptions about access to medical care may not hold in all areas of the world. We have therefore added the following text in the beginning of the subsection “COVID-19-related mortality” at line 268:

We assumed that all COVID-19-related deaths occur after hospitalization--that is, individuals do not die before being hospitalized. This assumption is likely valid for most developed countries; however, this assumption could be violated in communities without easy access to hospital facilities.

Line 313: “her” - maybe use a gender neutral verb 'their'

We have revised the text as suggested by the reviewer.

Line 365-367: it may be that low risk individuals don't have enough autonomy here. For example, even though I am a low risk individual, I would not meet with someone who tested positive (assuming that I find out, either through them telling me or social media, etc), so perhaps an extra term should be included to account for that

We acknowledge the reviewer's point that more flexibility in the probability of interaction for low-risk individuals would be an improvement to the model. However, the interaction rates are averages, so while there are certainly low-risk individuals who strongly reduce their interaction rates, there are other individuals who don't reduce their socialization at all. The addition of increased flexibility requires an increase in the number of model parameters and the complexity of the model. Therefore, to keep the model both manageable and understandable, we chose to base the interaction rates on average rates rather than allowing for increased individual flexibility.

Moreover, the individuals with a positive test reduce their contacts at a high rate of $r^{positive}$ in U(0.8,1), in addition to the already-reduced activity levels due to symptoms. This implies a very strong overall contact reduction of individuals with a positive test result.

Line 367: Rewrite equation as written on line 312 before adding the (1 - rsymptoms)

We agree with the reviewer. Using the same example individuals at both lines is probably easier for a reader. We have revised the text as suggested by the reviewer.

Line 368: Model analysis
The detailed description of the model is good given all the moving parts However, the authors should write out the detailed model for clearer understanding of the model either in the main text or as a supplementary material.

We interpret this comment as asking for a statement of the difference equations governing model dynamics. We acknowledge that in many studies a listing of the governing equations is helpful in understanding how the model works. However, in our case the model is complex enough that we don't feel an explicit statement of the governing equations would be helpful (it would indeed be very lengthy), and we rely on the detailed model description in the Methods section and the freely available source code (see our response to the comment just below).

Line 411: It will be helpful to readers if the authors can provide their source codes or a link to the source codes.

A statement of data availability will be included with the final published article. The complete Python implementation of the model is available at Github at https://github.com/ckadelka/COVID19-network-model. We added this sentence to the Acknowledgments.

S2-3 Fig: The y axis could be relabeled to something a bit more informative, like disease incidence

We thank the reviewer for the comment. As these two figures show collections of histograms for twelve secondary model parameters and outcomes, we believe "count" is the correct label for the y-axis. The only other option would be to have subplot specific y-labels but all information needed to distinguish the sub-plots is already provided by the specific x-labels.

S1 Table: Consider adding parameter descriptions with the table

We thank the reviewer for this suggestion. We added parameter descriptions to each row in S1 Table.

-Condensing the explanations for the supplementary information in the pdf files would have been helpful

We are unfortunately uncertain what the reviewer is referring to here. We believe this may be related to the PLOS-specific formatting of the manuscript (e.g., making each supplement a separate downloadable file), which we cannot change.

Reviewer #2: This is a very comprehensive study with some valuable insights. However, the amount of different policy domains touched on sometimes muddles some key points for that deserve more emphasis. For example, on pg. 3, consider the sentence "Furthermore, within each risk-group, testing recently-infected individuals first was more effective than prioritizing individuals who have been infected longer." This statement is fairly obvious and might not be a feasible policy choice. This sentence is either not needed or there should be more emphasis on the finding about superior results for public vs private contact reduction along with difference in benefits based on network structure. Overall this is a good paper that can be strengthened by emphasizing and expanding on key results, along with a few minor edits based on comments below.

We agree with the reviewer that our findings about testing policy are intuitive, although we are not sure that they are obvious. Given the current climate of uncertainty and confusion surrounding COVID-19 control policies, we feel that our testing results have both social and scientific value and should be reported. We agree with the reviewer that the results related to public vs private contact reduction may be more interesting, and we have added a sentence to emphasize this result to the Conclusion:

[Line 193] Our results support current policies to contain the outbreak and suggest possible refinements to public health policy and education. Specifically, because a reduction in public contacts more effectively controls disease spread than an equal reduction in private contacts, future public health notices could emphasize the need to reduce contacts specifically with random individuals. Our results also provide a possible explanation for why some societies are more successful at containing the outbreak despite the implementation of similar policy measures.

Specific comments:

1. On pg. 2 line 10: Authors state "to our knowledge there are no studies examining several policy domains simultaneously". This is a very naive statement given the amount of research

conducted on COVID-19. One example, among several others: https://doi.org/10.1101/2020.06.10.20127860

We thank the reviewer for drawing our attention to this new study. We have re-reviewed the literature, and edited the manuscript to include citations to several recent papers on this topic (lines 11-12), including the one suggested by the reviewer. We are happy to see more researchers investigating this important area.

2. pg. 3 line 60: "Our model yielded epidemiological outcome measures within the range of current estimates [3, 4, 18]: an average initial basic reproductive number (R0) of 2.76 and an average disease generation time of 5.29 days." The use of the word yielding is potentially misleading or unclear since R0 and generation time really depend directly on the input parameters.

We thank the reviewer for this comment, and have revised the text accordingly:

~~Our model yielded epidemiological outcome measures~~ The epidemiological outcome measures from our model fall within the range of current estimates…
